# Spatial Relationships between GABAergic and Glutamatergic Synapses on the Dendrites of Distinct Types of Mouse Retinal Ganglion Cells across Development

**DOI:** 10.1371/journal.pone.0069612

**Published:** 2013-07-26

**Authors:** Adam Bleckert, Edward D. Parker, YunHee Kang, Raika Pancaroglu, Florentina Soto, Renate Lewis, Ann Marie Craig, Rachel O. L. Wong

**Affiliations:** 1 Graduate Program in Neurobiology and Behavior, University of Washington, Seattle, Washington, United States of America; 2 Department of Biological Structure, University of Washington, Seattle, Washington, United States of America; 3 Department of Ophthalmology, University of Washington, Seattle, Washington, United States of America; 4 Psychiatry, Brain Research Center, Vancouver, British Columbia, Canada; 5 Transgenic Vector Core, Washington University, St. Louis, Missouri, United States of America; Institut National de la Santé et de la Recherche Médicale (INSERM U901), France

## Abstract

Neuronal output requires a concerted balance between excitatory and inhibitory (I/E) input. Like other circuits, inhibitory synaptogenesis in the retina precedes excitatory synaptogenesis. How then do neurons attain their mature balance of I/E ratios despite temporal offset in synaptogenesis? To directly compare the development of glutamatergic and GABAergic synapses onto the same cell, we biolistically transfected retinal ganglion cells (RGCs) with PSD95CFP, a marker of glutamatergic postsynaptic sites, in transgenic *Thy1­YFPγ2* mice in which GABA_A_ receptors are fluorescently tagged. We mapped YFPγ2 and PSD95CFP puncta distributions on three RGC types at postnatal day P12, shortly before eye opening, and at P21 when robust light responses in RGCs are present. The mature I_GABA_/E ratios varied among ON-Sustained (S) A-type, OFF-S A-type, and bistratified direction selective (DS) RGCs. These ratios were attained at different rates, before eye-opening for ON-S and OFF-S A-type, and after eye-opening for DS RGCs. At both ages examined, the I_GABA_/E ratio was uniform across the arbors of the three RGC types. Furthermore, measurements of the distances between neighboring PSD95CFP and YFPγ2 puncta on RGC dendrites indicate that their local relationship is established early in development, and cannot be predicted by random organization. These close spatial associations between glutamatergic and GABAergic postsynaptic sites appear to represent local synaptic arrangements revealed by correlative light and EM reconstructions of a single RGC's dendrites. Thus, although RGC types have different I_GABA_/E ratios and establish these ratios at separate rates, the local relationship between excitatory and inhibitory inputs appear similarly constrained across the RGC types studied.

## Introduction

Proper circuit function depends on the balance of excitation and inhibition on individual neurons as well as across their network. This balance likely needs to be regulated not only across the cell, but also locally, at the level of dendritic branches and spines. This is because the spatial distributions of synaptic inputs on some neurons [1], [2] appear highly organized. For example, functionally distinct synapses contact either the apical or basal dendritic segments of pyramidal neurons in somatosensory cortex [3]. Also, *Xenopus* retinal axons map topographically onto the dendrites of tectal neurons [4]. Such specific patterns of excitatory inputs also suggest that different dendritic segments may have distinct ratios of inhibitory to excitatory synapse densities (I/E ratio). Indeed, the I/E ratio varies across hippocampal neuronal arbors [5]. I/E ratios may be regulated at an even more local level. For instance, inhibitory and excitatory inputs in close proximity are more likely to be co-regulated by alterations in activity [6], [7]. Moreover, I/E ratios can show considerable variation for different cell types in the adult [8].

How I/E ratios are established during development, across the dendritic arbor of a cell, and along local dendritic segments remains unresolved. To gain insight, it is necessary to map both synapse types throughout the dendritic arbor of the same cell. The compact circuitry of the retina [9] makes this tissue particularly useful for mapping the connectivity of individual cells during development and at maturity [10], [11]. The extensive classification of mouse RGCs [12]–[14] further enables comparison of the I/E distributions and ratios for morphologically and functionally distinct RGC types [9].

To map inhibitory postsynaptic sites, we previously generated a transgenic line [15] in which Neuroligin2 (NL2), a transynaptic adhesion molecule at inhibitory synapses [16], is fused to YFP. However, NL2 is present at both retinal GABAergic and glycinergic synapses [15], [17]. Here, we directly compared the distributions of a defined set of inhibitory (GABAergic) synapses with that of glutamatergic synapses on the same RGC, by generating a new transgenic line in which expression of the γ2 subunit of GABA_A_ receptor, fluorescently tagged with YFP (*Thy1-YFPγ2*), is driven by the *Thy1* promoter. We biolistically transfected RGCs in retinas from these mice with fluorescently tagged PSD95, marking excitatory postsynaptic sites, in order to simultaneously visualize GABAergic and glutamatergic synapses throughout the dendrites. We compared the arrangements of both synapse types across three RGC types at two key developmental ages: Postnatal day (P)12, shortly after the onset of excitatory synaptogenesis [18] but before eye opening, and at P21, when synaptic densities within the inner retina reach their mature levels [18], and RGCs have robust light responses [19], [20]. We measured the distances between nearest neighbor synaptic sites and asked whether local distributions of GABAergic and glutamatergic synapses on the RGC dendrites exhibit any spatial constraints. Finally, we correlated fluorescently-labeled postsynaptic sites with amacrine and bipolar cell synapses by performing sequential light and EM reconstructions on the same cell. We were able to determine that while individual RGCs can have unique ratios of their total GABAergic and glutamatergic synapses at maturity, they establish these ratios at different times during development. However, the local spatial relationships between GABAergic and glutamatergic synapses on the dendrites of each type of RGC examined appeared similarly constrained from early in development.

## Materials and Methods

### Ethics statement

This study was conducted with the approval of the University of Washington Institutional Animal Care and Use Committee (Protocol 4122-01) and the University of British Columbia Animal Care Committee (Protocol A09-0278). Mice were euthanized by isoflurane overdose followed by decapitation (Protocol 4122-01), or they were euthanized by CO_2_ overdose (Protocol A09-0278).

### Mice

*Thy1-YFPγ2* mice were generated from cDNA for rat GABA_A_ receptor γ2 subunit (GABA_A_R γ2), short form, containing hexahistidine and EYFP near the mature N-terminus [21] and cloned into the Thy1 vector [22]. Mice of both sexes were used.

### Tissue Preparation

Mice were euthanized and enucleated and the eyes immersed in oxygenated mouse artificial cerebral spinal fluid (mACSF) containing the following in (mM): 119 NaCl, 2.5 KCl, 2.5 CaCl_2_, 1.3 MgCl_2_, 1 NaH_2_PO_4_, 11 glucose, and 20 HEPES, and brought to pH 7.42 with NaOH. To prepare retinal slices, the lens and vitreous were removed and the eye cups then lightly fixed in 4% paraformaldehyde in mACSF for 5 min. The sclera was then pealed away to isolate the retina, and the tissue was subsequently post-fixed for 15–20 min at room temperature. The retinas were rinsed in PBS pH 7.42 and mounted in 4% Type VII-A Agarose (Sigma), and sliced into 60 µm sections using a vibratome. To obtain retinal whole mounts, retinas were isolated in mACSF and mounted flat, ganglion cell side up, onto filter paper (Millipore).

### Biolistic transfection

Plasmids for which a cytomegalovirus promoter drives expression of tandem dimer Tomato (tdTomato) or postsynaptic density protein 95 fused to cyan fluorescent protein (PSD95CFP) were coprecipitated onto gold particles (Bio-Rad) [23]. Gold particles were propelled into whole mount retinas using a Helios Gene Gun (Bio-Rad), and the tissue then incubated at ∼34°C in oxygenated mACSF in a humidified chamber for 24 hr. Afterwards, retinas were fixed in 4% paraformaldehyde in mACSF for 20–30 min, rinsed in PBS, and flat mounted in vectashield (Vector Laboratories) for confocal imaging.

### Immunohistochemistry

Retina slices or flat mounts were incubated with primary antibodies in PBS for 4 days at 4°C. We used antibodies against gephyrin (mAb7a, SYSY, 1∶500 ∼mouse IgG Cat#147 011), PSD95 (ab2723, Abcam, 1∶1000 ∼mouse IgG2a, Cat#6G6-1C9), GABA_A_R α1 (gift of JM Fritschy 1∶5000 ∼rabbit, 1∶5000 ∼guinea pig), GABA_A_R α2 (gift of JM Fritschy 1∶2000 ∼guinea pig), GABA_A_R α3 (gift of JM Fritschy 1∶3000 ∼guinea pig), and GABA_A_R γ2 (gift of JM Fritschy 1∶4000 ∼guinea pig). Retinas were then washed and incubated with secondary antibodies, Alexa-568 or 633 (Invitrogen, 1∶1000), or DyLight-594 or 649 (Jackson Laboratory, 1∶1000) overnight at 4°C. Staining of cell nuclei was accomplished by incubating the retinas in To-Pro-3 (Invitrogen, 1∶4000) for 15 min at 24°C.

### Western blot

Brain tissue was collected from adult mice and homogenized in buffer (50 mM NaH_2_PO_4_, 300 mM NaCl, pH 8.0) with protease inhibitor cocktail (Roche), and centrifuged at 1500×g for 10 min at 4°C. The supernatant was collected and the protein concentration determined with the Bio-Rad Protein Assay kit. Western blot was performed directly with equal amounts of homogenate or after solubilization with 0.5% n-Dodecyl β-D-maltoside or 0.75% NP40 with equivalent results. Samples were run on SDS-polyacrylamide gels (10%), transferred to membranes, and blocked with 5% skim milk in Tris-buffered saline/0.05% Tween-20. Membranes were incubated with primary (anti-GABA_A_R γ2, Novus Biologicals NB300-190 or anti-GFP, Rockland 600-401-215) and secondary (HRP conjugated, Millipore) antibodies, and signal was detected using the SuperSignal Chemiluminescent kit (Thermo Scientific).

### Image Acquisition

Image stacks were acquired on an Olympus FV-1000 laser scanning confocal microscope with an oil-immersion 60x objective (Olympus, 1.35 NA). Voxel dimensions were (x-y-z in µm) 0.103-0.103-0.3 for images of single RGCs in whole mount retinas, 0.082-0.082-0.3 for images of retina slices, and 0.101-0.101-0.4 for correlative fluorescence and electron microscopy. Images were median filtered to remove noise and corrected for xy drift if necessary, and then compressed to 8-bits for analysis after normalization of the entire stack histogram using Fiji [24].

### Image Analysis

#### Cross-Correlation Analysis

To characterize the fluorescence expression within the inner plexiform layer (IPL) of retinas from *Thy1-YFPγ2* mice, we wrote custom MATLAB (Mathworks) scripts to calculate the cross-correlation coefficient between two channels of fluorescence (‘red’ and ‘green’) as described previously [15]. Briefly, for two image stacks, the correlation coefficient between every ‘red’ voxel and every corresponding ‘green’ voxel was calculated using the corrcoef function of MATLAB. We could determine if this observed correlation has a spatial constraint by shifting one channel incrementally up to 4 µm (within the xy plane of the retina) with respect to the other channel. A peak at the origin in the resulting 2D correlogram suggests that there is a shared spatial pattern between the two channels. We could further assess if the observed correlation is non-random by rotating one channel by 180° with respect to the other before shifting. A lack of peak correlation under this condition suggests that the observed correlation is not due to random distributions of fluorescence.

These measures examined the correlation within the entire IPL neuropil, however the retina IPL consists of several sublaminae which have unique distributions of cell types and synaptic profiles [25]. Thus, we divided the entire depth of the IPL into 100 percentiles and calculated the correlation coefficient between the two channels within each percentile. We also calculated the normalized fluorescence intensity for each channel (sum of intensities within each percentile/maximum sum of intensities within a percentile). When imaging two separate fluorescence channels, it is possible there will be differences in the intensities as well as signal to noise between the two channels. To determine how robust cross-correlation analysis is to these potential variations in fluorescence, we examined the cross correlation throughout the depth of a small volume of immunostained retina IPL with its duplicated volume (Figure S1). To test how differences in intensities affect correlation, the correlation coefficient was calculated after the histogram of the duplicated volume was linearly scaled (by 0.99, 0.95, 0.90, 0.75, 0.50, 0.25, 0.10, 0.05, 0.01). To test how signal to noise affects the correlation, the correlation coefficient was calculated after varying percentages (1, 5, 10, 25, 50, 75, 90, 95, 99) of voxels in the duplicated volume were randomly chosen and their intensities were randomly reassigned.

#### Quantification of RGC dendrites

The branching patterns of RGC dendrites were skeletonized and volume rendered using the filament function of Imaris (Bitplane). Total dendritic length was calculated from the skeletonized filament. The dendritic territory was calculated by expanding a z projection of the dendritic skeleton with a 10 µm diameter disk [26]. ON-S and OFF-S A-type RGCs were identified based upon their characteristic arbor morphologies [13], [14], [27], [28] and stratification within the IPL (Figure S2). Bistratified RGCs were assumed to be direction selective (DS) based upon their dendritic morphology and by their stratification within the densely labeled sublaminae of *Thy1-YFPγ2* expression that strongly correlated with VAChT (Figure S2) and GABA_A_R α2 labeling [29], [30]. Although previous studies of mouse RGC maturation did not find significant differences in RGC development with retinal eccentricity [27], we avoided RGCs from the inner and outer 10% of the retina.

#### Identification and quantification of postsynaptic puncta

The dendritic volume was expanded (xy 1.0 µm, z 0.5 µm) and used as a mask to segment Thy1-YFPγ2 fluorescence within and juxtaposed to the dendrites. Potential PSD95CFP and YFPγ2 puncta within this masked volume were identified using custom MATLAB scripts previously described [26]. Briefly, fluorescence signals within each channel were iteratively thresholded, filtered by size and contrast, and their ratio of fluorescence to cytosolic fluorescence intensity. In addition, puncta contacting the edge of the expanded mask were removed, and a final user-guided error minimization step was conducted by manual identification of the found puncta in 3D using the spots function of Imaris (Bitplane). Colocalization of puncta was classified by the sharing of at least one voxel between each identified puncta in one channel with those identified in the second channel.

#### I/E density maps and Sholl analysis

Density heat maps of puncta were calculated by dividing the total number of found puncta by the total dendritic length of the skeletonized dendrite within a 20 µm diameter sliding window along the dendrites [26]. Sholl analysis [31] of the I/E ratio was calculated by dividing the total number of YFPγ2 by the total number of PSD95CFP puncta within a sliding 10 µm window from the cell body. To exclude the cell body the initial 10 µm were omitted.

#### Nearest neighbor analysis

The nearest neighbor 3D distances were calculated for every found puncta on each RGC for three different groupings: Each PSD95CFP and the nearest PSD95CFP, each YFPγ2 and the nearest YFPγ2, and each PSD95CFP and the nearest YFPγ2. The median nearest neighbor distances were calculated for each group, as well as the cumulative distributions for each RGC. To determine if the median distances obtained between found puncta were non-random, we simulated a sample population of P12 and P21 RGCs with random distributions of PSD95CFP and YFPγ2 puncta on their dendrites. We utilized the dendritic volumes of one P12 and one P21 RGC of each type (ON-S A-type, OFF-S A-type, and bistratified DS) as our model RGCs. To create a sample of cells that matched our observed RGC distributions we utilized the total linear densities of PSD95CFP and YFPγ2 puncta obtained from P12 and P21 RGC to calculate the total numbers of puncta to be distributed across the dendritic volume. The total number of puncta for each simulated RGC was calculated by multiplying the linear densities by the total dendritic length of the model RGCs. These total numbers of puncta were then randomly distributed within the volume of the model RGCs with the constraints that no PSD95CFP puncta could occupy the same voxel coordinate of another PSD95 puncta, and that no YFPγ2 puncta could occupy the same voxel coordinate of another YFPγ2 puncta. Using these parameters, the median nearest neighbor distances between puncta groups and cumulative distributions were calculated for model ON-S A-type P12 (n = 9) and P21 (n = 9), OFF-S A-type P12 (n = 5) and P21 (n = 5), and bistratified DS P12 (n = 7) and P21 (n = 5) RGCs.

### Correlative Fluorescence and Electron Microscopy

For one ON-S A-type RGC at P21 we reconstructed the synaptic connectivity of bipolar cells and amacrine cells to the RGC after mapping the distributions of PSD95CFP and YFPγ2 puncta onto its dendritic arbor. A *Thy1-YFPγ2* mouse retina was biolistically transfected as described above. After incubation for 24 hrs, the retina was fixed in 1.5% glutaraldehyde/2.5% paraformaldehyde for 40 min. The tissue was washed and mounted under coverglass in 0.1 M sodium cacodylate buffer for confocal and multiphoton imaging. An image stack of the labeled RGC was obtained for one quadrant of the dendritic arbor including the cell body. The retina was unmounted and fixed in 4% glutaraldehyde overnight. The retina was again washed and remounted in 0.1 M sodium cacodylate buffer. Using a custom-built two-photon microscope with a Ti:sapphire laser (Spectra-Physics) tdTomato fluorescence of the RGC was visualized and fiduciary marks were burned into the tissue using the near-infrared branding (NIRB) method [32]. A window was branded around the cell body and dendrites at the level of the soma, and a mark was branded through the depth of the IPL onto one side of the window. These marks were clearly evident as autofluorescence under multiphoton imaging and by the absence of tissue under electron microscopy.

An image stack of the RGC and autofluorescence of the burn marks was taken, and the retina was unmounted and a small section of retina encompassing the RGC was sectioned (∼1 mm^2^). This tissue block was then washed in 0.1 M sodium cacodylate buffer and post-fixed for 45 min in cacodylate buffered 1% osmium tetroxide. The tissue was washed in dH_2_O, en bloc-stained in 1% aqueous uranyl acetate, and washed again in dH_2_O before being dehydrated through a series of ethanol and propylene oxide and embedded in Epon Araldite resin. Semi-thin resin sections were cut until the burn marks could be identified in Richardsons stain (Methyl Blue Azure II) tissue sections. Serial thin sections (80 nm) were cut, placed on Formvar-coated slot grids and stained with Reynolds lead citrate. Micrographs were taken at 2200x (scanned at 2500dpi, ∼8.5 nm/pixel) for image registration, and 8900x (scanned at 2500dpi, ∼1.1 nm/pixel) for identification of synaptic contacts.

The EM micrographs were manually aligned and serially registered using TrakEM2 [33]. Bipolar and amacrine cell synaptic connections onto the RGC dendrites were identified using established criteria [34]. Briefly, bipolar cell terminals and sites of contact were identified by the presence of a presynaptic ribbon apposed to a postsynaptic density (PSD). Amacrine cell contacts were identified by the presence of a PSD and presynaptic vesicles. Often, amacrine contacts also contained dense core vesicles. The presynaptic terminals of bipolar and amacrine cells and the RGC dendrite were traced through successive serial sections to examine local circuit connectivity. Feedforward connections were identified when a bipolar cell contacted a RGC and an amacrine cell at a dyad synapse, and the amacrine cell subsequently contacted the RGC dendrite [35]. To correlate the identified fluorescent puncta with amacrine and bipolar cell synapses, a maximum intensity projection of the labeled RGC dendrite was scaled and aligned to the EM reconstruction using TrakEM2. Each identified synaptic contact was scored based on the presence or absence of a corresponding fluorescence marker, and vice versa, within the local dendritic segment reconstructed. Because the EM sections were ∼80 nm, it is possible that we did not detect some postsynaptic densities parallel to the plane of section, and thus our estimation of correlation is likely a lower bound [36].

### Statistics

When data passed tests of normality (Lillifors, Jarque-Bera), we utilized student's t-test. Otherwise we carried out the non-parametric Wilcoxon rank-sum test to determine significance. All other statistics are noted within the text.

### Data availability

All data presented in this manuscript are freely available upon request by contacting the corresponding author.

## Results

### Expression of YFPγ2 in the retina of *Thy1-YFPγ2* mice

**Figure 1:**
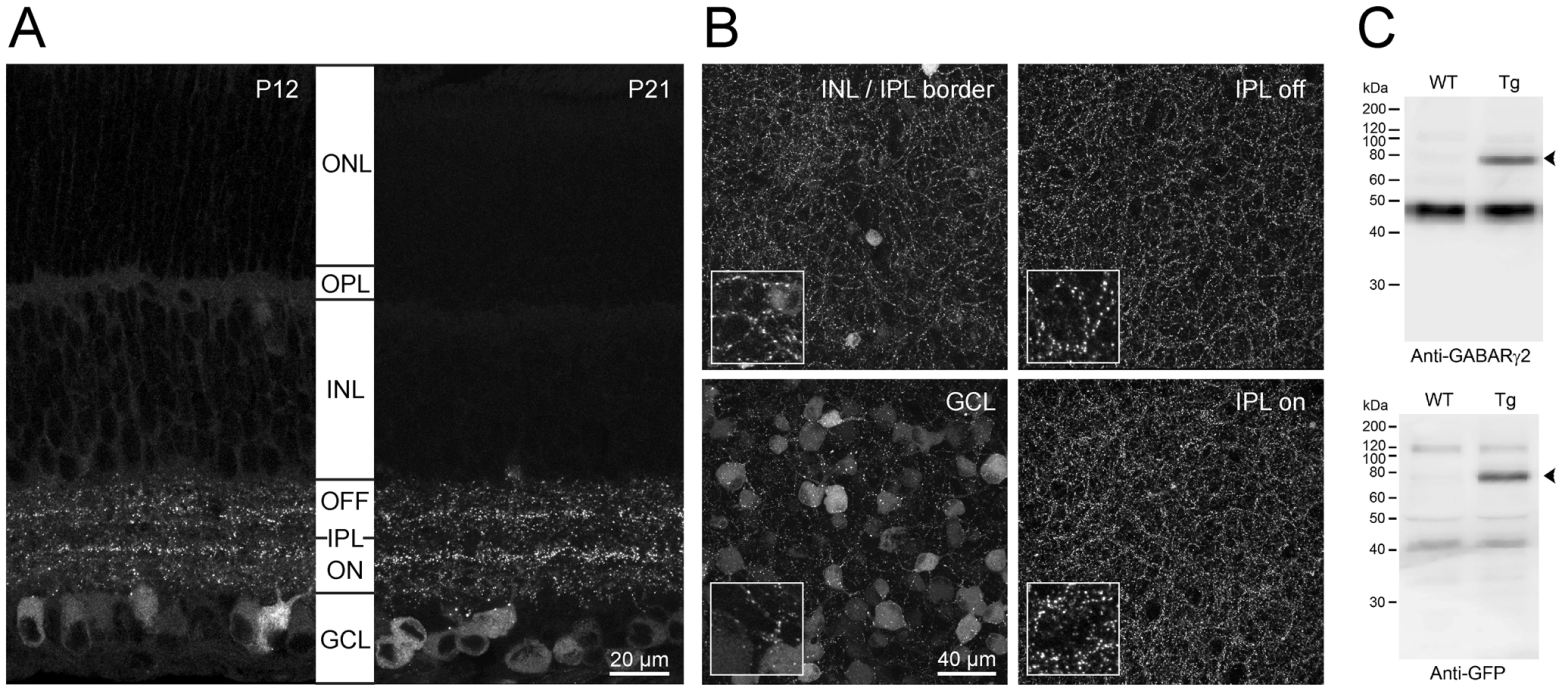
Pattern of YFPγ2 expression in retinas of *Thy1-YFPγ2* transgenic mice. (**A**) Maximum intensity projections (MIP) of confocal image stacks (2 µm total thickness) of vertical slices of *Thy1-YFPγ2* retinas at postnatal day (P)12 and P21. Diffuse fluorescence expression in cell bodies within the ganglion cell layer (GCL) at both ages, and punctate expression throughout both OFF and ON layers of the inner plexiform layer (IPL) are apparent. There was no expression in the outer plexiform layer (OPL) or the outer nuclear layer (ONL) at either age. (**B**) Images of MIPs (∼20 µm thick) at various depths of a P21 flat mount *Thy1-YFPγ2* retina. Inserts are 3× magnification of the images. (**C**) Western blot of whole brain lysates from *Thy1-YFPγ2* (Tg) and wildtype (WT) mice. YFPγ2 (arrowhead) was detected with anti-GABA_A_Rγ2 (upper) and anti-GFP (lower).

YFPγ2 expression was present as early as postnatal day (P)7 in the retina of *Thy1-YFPγ2* mice, but the labeling was diffuse and the signal to noise too low to quantify synaptic sites (data not shown). By P12, punctate fluorescence was consistently observed throughout both the ON and OFF sublaminae of the inner plexiform layer (IPL) although diffuse fluorescence was still visible in cell bodies of the ganglion cell layer (GCL) (Figure 1A). Although cell bodies remain labeled at P21, punctate labeling is more pronounced in the IPL. At both ages, the relative intensity and density of fluorescent puncta varies across layers in the IPL, with the highest density of expression localized to two substrata in the IPL (Figure 1A, B). Furthermore, expression was not observed in other layers of the retina at either age, except in a few cell bodies in the innermost layer of the inner nuclear layer (INL). The scarcity of cell bodies labeled in the INL compared to the GCL (Figure 1A, B), suggests that the majority of cells labeled in the *Thy1-YFPγ2* transgenic retina are RGCs. However, only ∼20% of cells in the GCL layer express YFPγ2 (Figure S3A–C), while 40% of the cells are likely RGCs [37], thus not all RGCs express YFPγ2 in the *Thy1-YFPγ2*. Likewise, not all puncta immunostained for the GABA_A_R γ2 subunit within the IPL also have YFPγ2 labeling, but all YFPγ2 puncta are labeled by anti- GABA_A_R γ2 (Figure S3D).

We quantified the level of expression of YFPγ2 in *Thy1-YFPγ2* mice by obtaining western blots of whole brain lysates (Figure 1C). We found that the overall expression level of the transgenic YFPγ2 was 31.5%±1.3, relative to endogenous GABA_A_R γ2 protein level (n = 4). The YFPγ2 mice appeared undistinguishable from wild-type with respect to viability, fertility, and home cage behavior. Previous transgenic lines expressing untagged γ2 showed that even with expression at higher level, roughly 100% relative to endogenous, mice exhibited only a difference in acute functional tolerance to ethanol but no other differences in multiple behavioral and biochemical tests [38].

### Cross-correlation analysis can detect overlapping spatial distributions between two fluorescent labels

We wished to determine how well fluorescence expression in *Thy1-YFPγ2* transgenic retina represents GABA_A_R distributions. To confirm correct localization of the punctate fluorescence in the *Thy1-YFPγ2* transgenic retina to sites of GABAergic synapses, we assessed the colocalization of YFPγ2 signal with immunostaining of proteins found at inhibitory postsynaptic sites. Because of variability in immunostaining and fluorescence expression, it is often difficult to systematize methods of colocalization across samples. Here we used correlation analysis because it is strongly insensitive to differences in intensity between two fluorescent channels, and can be used to detect spatial correlations between two image volumes (Figure S1) [15], [39].

**Figure 2:**
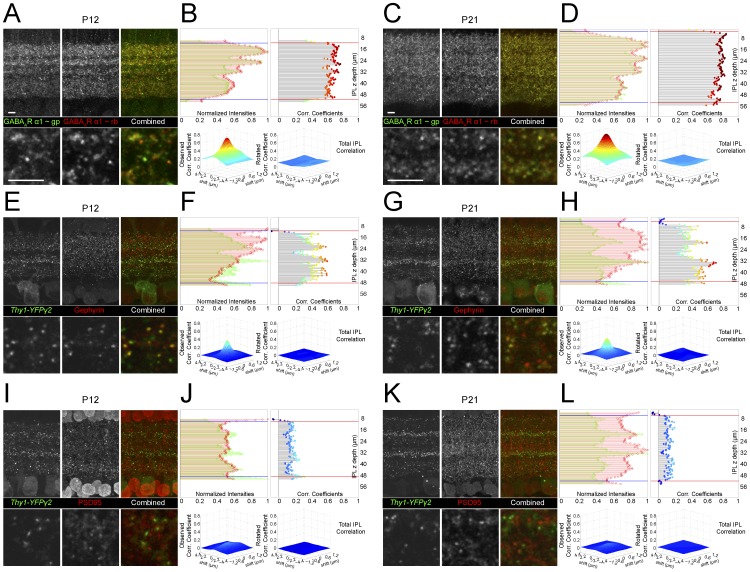
YFPγ2 fluorescence correlates with immunostaining of inhibitory postsynaptic, but not excitatory postsynaptic sites. (**A, C**) Vertical sections from P12 and P21 retinas immunostained with anti-GABA_A_R α1 raised in different hosts. Upper panels are MIPs of image stacks of 15 µm total thickness. Lower panels show single optical sections (0.3 µm) within the stack at higher magnification. (**B, D**) (Upper-Left) Normalized fluorescence intensities for GABA_A_R α1 ∼gp (green) and GABA_A_R α1 ∼rb (red) are plotted against the vertical (z) depth of the IPL. (Upper-right) The correlation coefficient obtained from the cross-correlation of the two fluorescent channels plotted against depth of the IPL. (Lower panels) 2D cross-correlation plots for the entire depth of the IPL (region between red lines in upper-right). Correlation between the two channels decreases when one channel is shifted in ‘x and y’ (left plot) away from 0,0 or if one channel is rotated 180° (right plot) with respect to the other. (**E–H**) Cross-correlation analysis was also performed for vertical sections from P12 and P21 *Thy1-YFPγ2* retinas immunostained with anti-gephyrin, a protein found at inhibitory postsynaptic sites. (**I–L**) Similar plots for the excitatory postsynaptic marker, PSD95 at P12 and P21. Strong correlation between *Thy1-YFPγ2* fluorescence and inhibitory, but not excitatory, postsynaptic markers is present early in postnatal development. Scale bars 5 µm.

We determined how well cross-correlation analysis can identify the spatial relationship between two separate fluorescence labels in retinal sections by obtaining the correlation profiles of a single protein visualized by distinct fluorescence signals found at inhibitory GABAergic synapses. To label a single protein with two fluorophores (568 and 649), we used primary antibodies against GABA_A_R α1 subunit that were raised in different hosts, guinea pig (∼gp) and rabbit (∼rb) (Figure 2A, C). We obtained two types of cross-correlation plots for both ages studied (P12 and P21): one that plots the observed correlation coefficient at various planes of IPL depth, and the other provides a 2D cross-correlogram of the two signals indicating how the correlation between the two channels is altered by changes in their relative spatial alignment. We found a strong correlation throughout the IPL for the two distributions of GABA_A_R α1 labeling, and the correlation coefficients at each sublamina are of similar magnitude (Figure 2B). The high degree of spatial similarity was also evident in single optical sections where the two signals overlap extensively (Figure 2A, C). To determine whether this observed correlation was due to random distributions of both fluorescence signals, we obtained a 2D cross-correlogram of the observed signals by shifting one channel (in x and y) with respect to the other. We found that for the entire IPL, the 2D cross-correlogram peaks at the observed spatial orientation (0,0), suggesting that the spatial correlation of the immuno-fluorescence from the two antibodies is not random (see Materials and Methods). This non-random correlation is also evident in the flat 2D correlogram obtained when one channel was rotated 180 degrees (in x and y) relative to the other (Figure 2B, D). Overall, the cross-correlation approach we use here is a reasonable quantitative method for assessing whether two signals are non-randomly associated with each other, and provides a metric for assessing the relative strength of spatial correlation between two channels across multiple types of fluorescence labeling.

### YFPγ2 expression correlates with immunolabeling of proteins at inhibitory but not excitatory postsynaptic sites

The endogenous γ2 subunit incorporates into GABA_A_R localized to synaptic sites [40] and is necessary for the clustering of GABA_A_R at synapses [41]. To determine whether exogenous expression of YFPγ2 in the *Thy1-YFPγ2* transgenic retina correctly localizes to GABAergic postsynaptic sites, we performed cross-correlation analysis of YFPγ2 fluorescence within the retina IPL immunostained for proteins known to be at inhibitory or excitatory synapses.

We first examined the distributions of YFPγ2 together with immunolabeling for gephyrin, a postsynaptic scaffolding molecule at GABAergic and glycinergic synapses [42] or PSD95, a postsynaptic scaffolding protein at glutamatergic synapses [43] (Figure 2E–L). At both ages, we found a strong correlation between gephyrin staining and YFPγ2 expression throughout the IPL (Figure 2F, H). This correlation is also evident in the degree of overlap of fluorescence puncta signals observed in single optical sections taken from image stacks (Figure 2E, G). However, the strength of correlation in each sublamina is not merely a result of the intensity of fluorescence, because the normalized intensities of each channel do not follow the same pattern across IPL layers (Figure 2F, H). Note that for gephyrin the observed correlation for the entire IPL (peak at 0,0 in 2D correlogram) is not as strong as the peaks in some individual sublamina because it reflects an average of all layers. In contrast, the pattern of correlation within the IPL for PSD95 shows weak correlation across all layers, despite the high density of PSD95 labeling (Figure 2I–L), and no overlap in fluorescence signal is apparent in single optical sections (Figure 2I, K). The strong correlation of YFPγ2 fluorescence with gephyrin and weak correlation with PSD95 immunostaining at both ages suggests that the *Thy1-YFPγ2* is reliably marking inhibitory and not excitatory postsynaptic sites within the IPL in the postnatal mouse retina.

### *YFPγ2* is present at GABAergic synaptic sites

**Figure 3:**
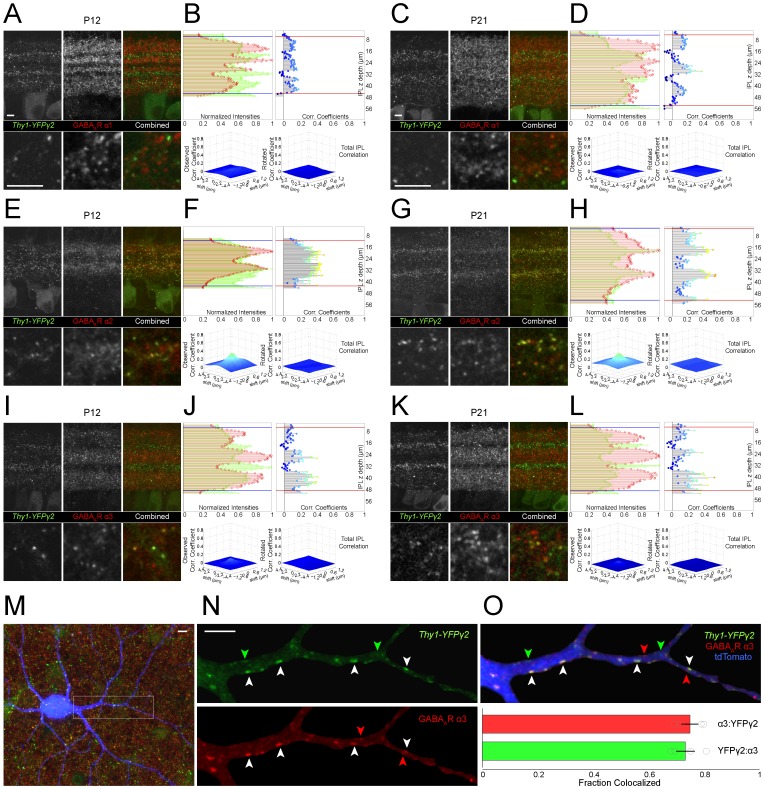
YFPγ2 fluorescence correlates with immunostaining for GABA_A_R α subunits. Cross-correlation analysis of vertical sections from P12 and P21 *Thy1-YFPγ2* retinas stained with anti-GABA_A_R α1 (**A–D**), GABA_A_R α2 (**E–H**), or GABA_A_R α3 (**I–L**). (**M**) Image stack of a single ON RGC labeled by transfection of CMV:tdTomato in a *Thy1-YFPγ2* retina, immunostained for GABA_A_R α3. (**N**) The dendritic label was used to mask out the YFPγ2 and GABA_A_R α3 fluorescence. (**O**) (Upper) YFPγ2 and GABA_A_R α3 puncta within sections of dendritic arbors identified by custom-written software. (Lower) Fraction of the population of YFPγ2 puncta that colocalized with GABA_A_R α3, and vice-versa (average and SEM of 4 cells). White arrowheads are examples of colocalized puncta, green and red arrowheads, non-colocalized puncta. Scale bars 5 µm.

Although it is clear that YFPγ2 fluorescence is associated with inhibitory synapses, gephyrin immunostaining alone does not distinguish between GABAergic and glycinergic synapses in the retina [44], [45]. To determine whether YFPγ2 is found within GABA_A_R clusters, we asked how well YFPγ2 fluorescence correlates with immunolabeled GABA_A_Rs. Because the γ2 antibody would label both endogenous γ2 and YFPγ2, immunostaining for this receptor subunit will not help determine whether YFPγ2 correctly localizes to GABAergic postsynaptic sites. We thus immunolabeled endogenous α1, 2, and 3 subunits (Figure 3) because these subunits are found in the majority of synaptic GABA_A_Rs [46].

In the retina, each GABA_A_R cluster bears only one type of α subunit [47]. We found that YFPγ2 colocalizes with GABA_A_R α1 (Figure 3A–D), GABA_A_R α2 (Figure 3E–H) and GABA_A_R α3 (Figure 3I–L). Peaks in the correlation with YFPγ2 occurred at different sublaminae in the IPL for each of the α-subunits. YFPγ2 expression appears to be most dense in two strata where GABA_A_R α2 receptors are concentrated, and the correlation coefficient is high between these two GABA_A_R labels. In contrast, there is less YFPγ2 expression in IPL strata that contain high densities of GABA_A_R α1 or GABA_A_R α3 labeling, most likely because fewer cells that predominantly express these α-subunits also express the transgene. It is therefore not unexpected that compared to α2, the correlation coefficient of YFPγ2 and the α1 or α3 signal is less pronounced (flatter correlogram), although still above chance. It is important to emphasize why not all immunolabeled GABA_A_Rs colocalize with YFPγ2 signal. YFPγ2 is primarily expressed by RGCs, but not by all RGCs (Figure 1, S3). Also, GABA_A_Rs are expressed by RGCs, amacrine cells and bipolar cells whose processes all contribute to the inner plexiform layer. But, in the transgenic line, YFPγ2 is expressed in very few amacrine cells and not in any bipolar cells. The antibody labeling for α subunits of the GABA_A_R will label all endogenous receptors in the retina including receptors in cells not expressing YFPγ2. We therefore determined how well YFPγ2 expression represents the total population of endogenous GABA_A_Rs on an individual RGC.

We mapped YFPγ2 and GABA_A_R α3 puncta on the dendritic arbors of one type of RGC that stratifies its dendrites within the ON layer of the IPL. We chose the ON-S A-type RGC for this analysis because the probability of obtaining a cell-fill by biolistic transfection in the *Thy1-YFPγ2* retina, in which not all RGCs express the transgene, is reasonably high. To co-map YFPγ2 and GABA_A_R α3 puncta, we biolistically transfected retinas in the *Thy1-YFPγ2* retina with CMV-tdTomato at P21 (Figure 3M). We generated skeletons of the dendritic arbors that were subsequently used to isolate fluorescent puncta within the dendrites (Figure 3N), and then identified postsynaptic puncta using custom-written software [26] (see Materials and Methods). We found that for individual dendritic segments (n = 4 cells), the fraction of identified YFPγ2 puncta that also contained GABA_A_R α3 was 73.3%±0.03, and the fraction of identified GABA_A_R α3 puncta associated with YFPγ2 was 74.9%±0.03 (Figure 3O). Thus, for individual RGCs, YFPγ2 is a reliable marker of GABA_A_Rs, and represents the majority of GABA_A_Rs on an individual RGC dendritic arbor. Furthermore, our observations suggest that the ON-S A-type RGC expresses primarily GABA_A_R α3.

### Distributions of PSD95CFP and YFPγ2 within individual dendritic arbors of distinct RGC types

**Figure 4:**
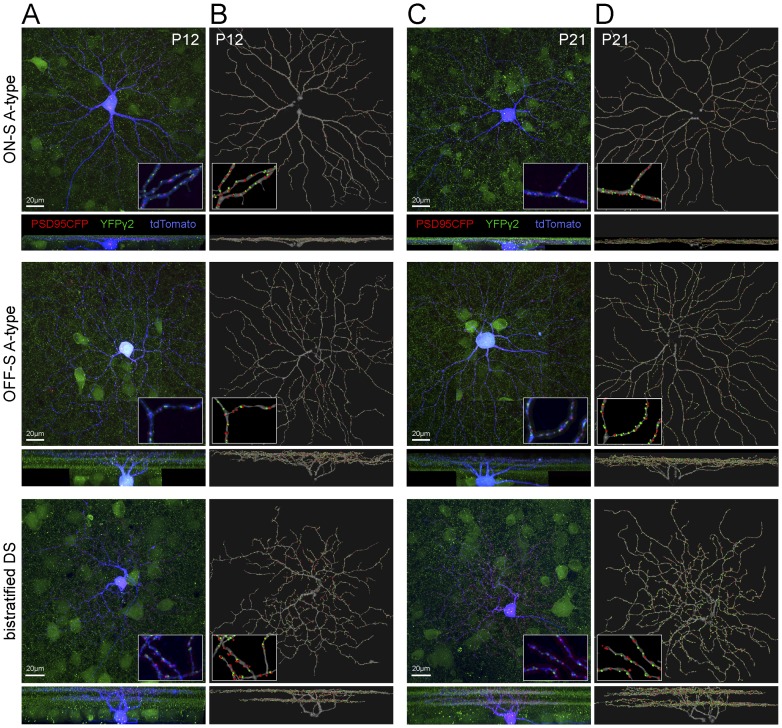
Distributions of GABAergic and glutamatergic postsynaptic sites on dendritic arbors of distinct types of RGCs. Biolistic transfection of individual ganglion cells in *Thy1-YFPγ2* retinas with a cell label (tdTomato) and excitatory postsynaptic protein (PSD95CFP). (**A**) Representative flat mount (upper) and vertical (lower) MIPs of three distinct RGC types in P12 *Thy1-YFPγ2* retina; ON-S A-type, OFF-S A-type, and bistratified DS RGCs. Inserts are 4x magnifications of a region of the arbor showing PSD95CFP and YFPγ2 found only within the dendrites. (**B**) MIPs of the reconstructed dendritic skeletons and identified postsynaptic sites from the cells in (A). Inserts represent the same regions as inserts in (A). (**C, D**) Example MIPs of the raw images of P21 cells (C), their skeletonized dendrites and identified postsynaptic sites (D).

**Figure 5:**
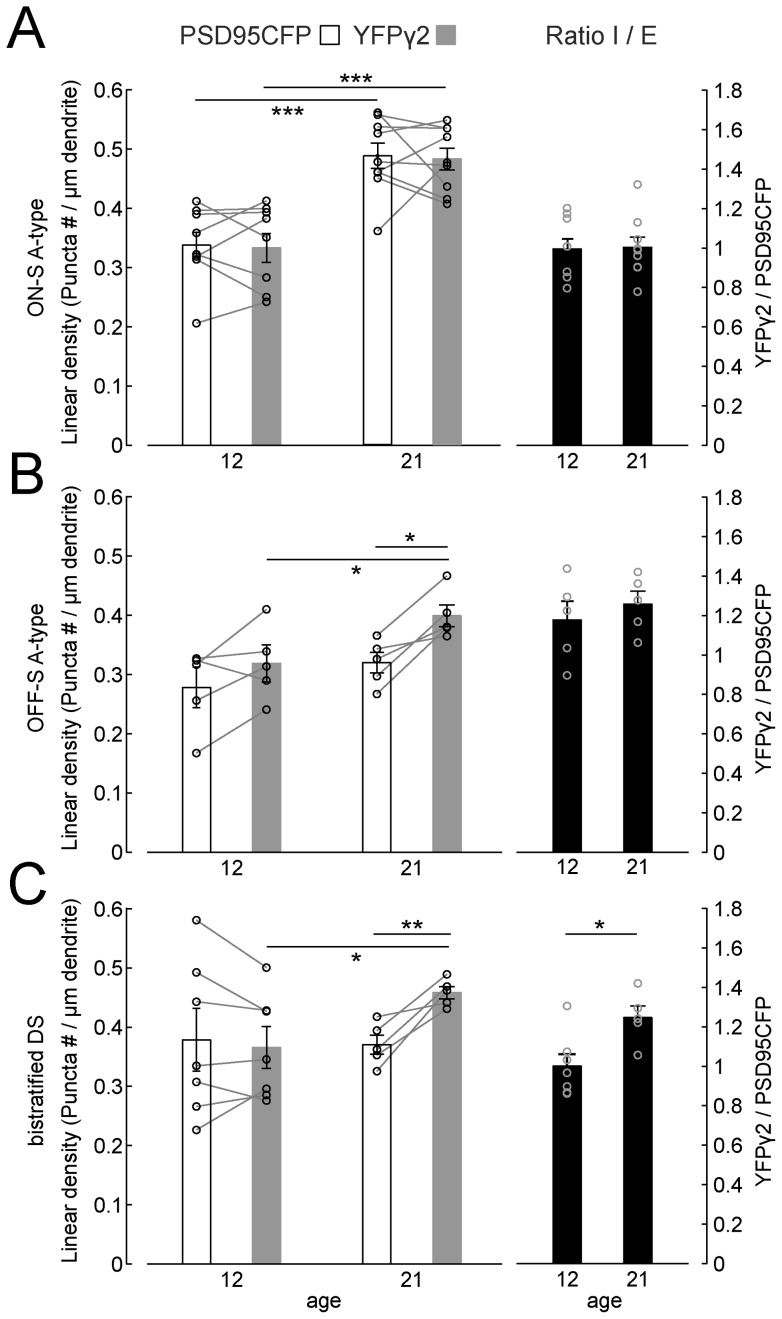
Densities and ratios of GABAergic to glutamatergic postsynaptic sites of three types of RGCs across development. Linear densities (left axis) and ratio (right axis) of PSD95CFP (white bars) and YFPγ2 puncta (gray bars) on P12 and P21 ON-S A-type cells (**A**), OFF-S A-type (**B**) and bistratified DS (**C**) RGCs. Lines link data points from the same cell. Statistical tests between ages were two sample student's t-test unequal variance, and within ages were two sample paired student's t-test. ***  =  P<0.001, ** =  P<0.01, *  =  P<0.05.

To map GABAergic and glutamatergic postsynaptic sites on individual RGCs, we biolistically transfected cells in the *Thy1-YFPγ2* retina with CMV-tdTomato and CMV-PSD95CFP (Figure 4A, C). We generated skeletonized dendritic arbors (Figure 4B, D) and used these to identify postsynaptic puncta [26] (see Materials and Methods; examples in Figure 4B, D). We quantified the total densities and ratios of YFPγ2 and PSD95CFP puncta on the dendritic arbors of each RGC at P12 and P21 by calculating the total numbers of puncta per dendritic length (Figure 5). We found that for ON-S A-type RGCs, the densities of both YFPγ2 and PSD95CFP increased from P12 to P21 (Figure 5A). However, their mature I/E ratio was already apparent at P12. Similarly, the mature OFF-S A-type RGC I/E ratio was attained by P12 (Figure 5B). However, at P21, OFF-S A-type RGCs I/E ratios are greater than that of ON-S A-type RGCs (p = 0.01 student's t-test). For bistratified DS cells, both inhibitory and excitatory synaptic densities showed greater variability at P12 (Coefficient of variation (CV), PSD95CFP  = 34.2, YFPγ2  = 23.9) than at P21 (CV, PSD95CFP  = 9.7, YFPγ2  = 5.0; Figure 5C). Although there was a clear increase in the density of inhibitory sites across ages, we did not detect a statistical change in the densities of excitatory synapses. It is evident that the ratio of I/E for each bistratified DS cell, and across all cells, increased between P12 and P21 (Figure 5C). This observation was true for both ON and OFF arbors of bistratified DS RGCs (Table 1). Although the I/E ratio at P21 for bistratified DS was greater than that of ON-S A-type RGCs (p = 0.01 student's t-test), there was no difference between the I/E ratios for OFF-S A-type and bistratified DS RGCs at this age. Taken together, mature I/E synapse density ratios, and rates at which these ratios are obtained during development, are different across RGC types.

**Table 1:** Summary data for total dendritic length, puncta linear densities, and total inhibitory to excitatory ratios for all RGCs sampled.

RGC type	Age	Dendritic Length (µm)	PSD95CFP number	YFPγ2 number	PSD95CFP linear density	YFPγ2 linear density	I/E ratio	n
**ON-S A-type**	12	3583.9±247.6	1185.2±71.4	1171.1±79.8	0.34±0.02	0.33±0.02	0.99±0.05	9
	21	3102.3±119.2	1534.6±102.3	1511.3±91.9	0.49±0.02	0.49±0.02	1.00±0.05	9
**OFF-S A-type**	12	4235.8±336.1	1163.8±172.0	1340.0±169.7	0.28±0.03	0.32±0.03	1.18±0.15	5
	21	4337.0±223.4	1382.4±96.0	1738.4±151.6	0.32±0.02	0.40±0.02	1.26±0.07	5
**Bistratified DS**	12	4028.0±246.8	1508.4±192.4	1454.0±118.9	0.38±0.05	0.37±0.04	1.00±0.06	7
ON arbor	12	2247.1±154.7	839.6±144.0	799.9±99.3	0.37±0.05	0.36±0.03	0.99±0.06	
OFF arbor	12	1780.9±179.8	668.9±80.6	654.1±57.4	0.39±0.05	0.38±0.04	1.01±0.07	
**Bistratified DS**	21	3766.4±165.6	1401.4±94.1	1729.8±60.3	0.37±0.02	0.46±0.01	1.25±0.06	5
ON arbor	21	2036.4±56.4	768.0±17.3	907.8±39.9	0.38±0.02	0.45±0.01	1.19±0.07	
OFF arbor	21	1730.0±170.0	633.4 ± 83.7	822.0±84.2	0.36±0.02	0.47±0.02	1.32±0.06	

Numbers are averages and SEM for all cells, for each parameter. Total dendritic length was calculated as the sum of all dendritic segment lengths within the (∼.045 mm^2^) region sampled.

It is possible that the decrease in nearest neighbor distances between PSD95CFP puncta and between YFPγ2 puncta in ON-S A-type RGCs with maturation, but not in the other RGC types, is, in part, due to the relatively greater increase in total synapse density in the ON A-type RGCs. For all RGC types studied, however, the median distance between neighboring PSD95CFP and YFPγ2 puncta is already established at P12, which cannot be accounted for by random distributions of GABAergic and glutamatergic synapse densities onto RGC arbors.

**Figure 6:**
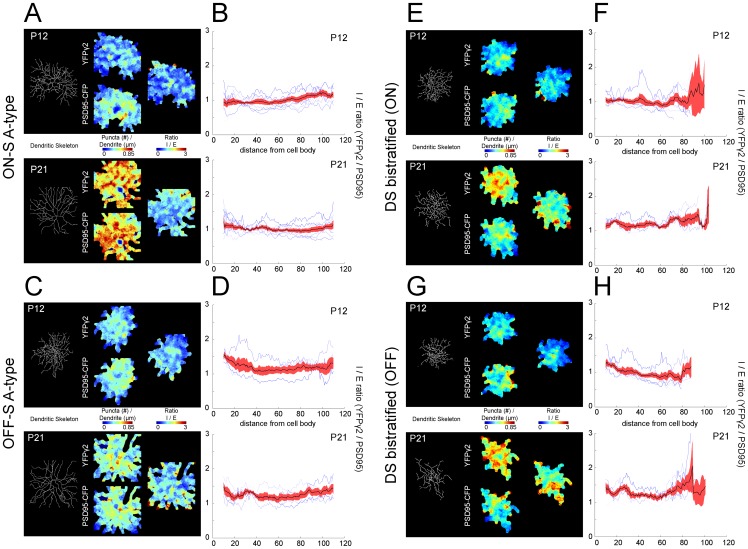
Spatial density heat maps of GABAergic and glutamatergic postsynaptic sites on RGC dendritic arbors. (**A**) (Left) Dendritic skeletons of ON-S A-type RGCs at P12 and P21. (Middle) Synaptic density maps were created by convolving a 10 µm disk centered on each pixel of the MIP of the dendritic skeleton puncta linear density [puncta (#)/dendrite (µm)] for YFPγ2 (upper) and PSD95CFP (lower). (Right) Ratio of YFPγ2/ PSD95CFP puncta across ages. (**B**) YFPγ2/ PSD95CFP ratio plotted against eccentricity (distance from cell body). Each blue line represents a cell, black line is mean and red shading SEM. Analysis of OFF-S A-type RGC is shown in (**C, D**) and of ON and OFF arbors of bistratified DS RGCs in (**E–H**).

It is known for cortical pyramidal neurons that the relative ratio of inhibitory to excitatory synapses is not evenly distributed across the dendritic arbor [5]. It is possible that the patterns of postsynaptic sites on RGCs similarly show differential distributions across their arbors [26], [48], [49]. We therefore mapped the densities of YFPγ2 and PSD95CFP and their ratios across the dendritic arbors of each of three RGC types (Figure 6). For all RGC types examined, the gradients for both YFPγ2 and PSD95CFP across the arbors were shallow. The I/E ratios of ON A-type RGCs appear evenly distributed across the dendritic arbor at both P12 and P21 (see example, Figure 6A). Similarly, OFF-S A-type RGCs showed an even I/E distribution across their arbors, also at both ages (Figure 6C, D). The density distributions for bistratified DS RGCs showed more variability for both ON and OFF arbors. While this variability appeared greater at the periphery of the dendritic arbor for some cells, we did not observe a systematic increase or decrease in the ratio of I/E in the periphery (Figure 6G, H).

### Local spatial relationships between inhibitory and excitatory synapses on RGCs are established early in development

Our experiments, thus far, considered how the densities of glutamatergic and GABAergic postsynaptic sites on RGCs alter with maturation. It has previously been suggested that dendritic remodeling and an increase in total synaptic number with maturation contribute to defining the adult densities of excitatory [26] and inhibitory [15] synapses on RGCs. To gain a sense of how developmental increases in glutamatergic and GABAergic synapse densities are reflected locally at neighboring synapses, we assessed the nearest neighbor distances between PSD95CFP puncta, between YFPγ2 puncta, and between PSD95CFP and YFPγ2 puncta, at P12 and P21.

**Figure 7:**
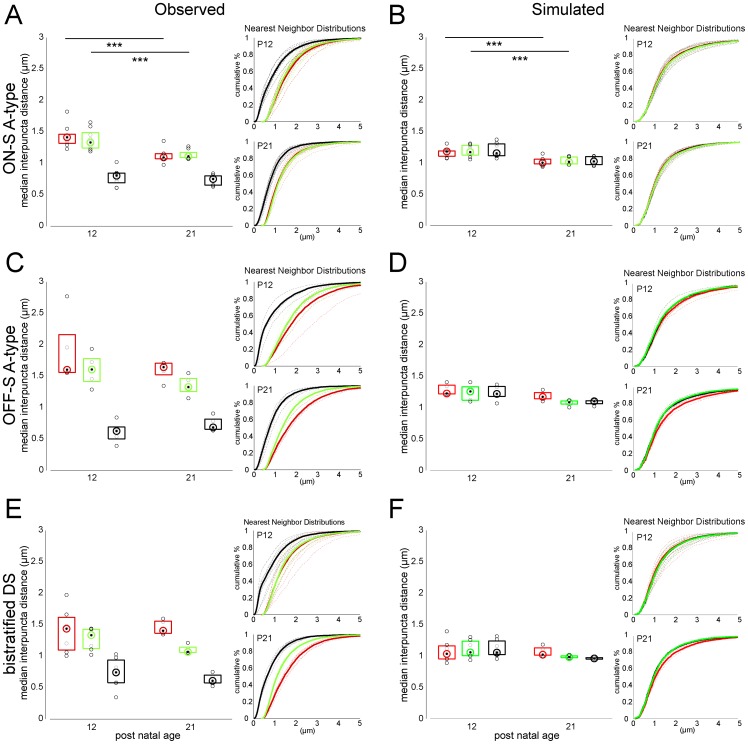
The nearest neighbor distance between PSD95CFP puncta and their nearest YFPγ2 punctum is established early in development. (**A**) Plotted are the median nearest neighbor distances for each PSD95CFP punctum and its nearest neighbor (red box plot), each YFPγ2 punctum and its nearest neighbor (green box plot) and each PSD95CFP punctum and the nearest YFPγ2 punctum (black box plot) for ON-S A-type RGCs at P12 and P21. (**B**) The nearest neighbor distances calculated from simulating the same densities of PSD95CFP and YFPγ2 puncta from ON-S A-type RGCs randomly distributed over their dendritic arbors. Nearest neighbor analysis was also performed for OFF-S A-type (**C–D**), and bistratified DS RGCs (**E–F**). Box plots represent 25 and 75 percentiles. Inserts show cumulative nearest neighbor distributions for P12 (upper) and P21(lower). Dashed lines are individual cells, solid lines represent all cells. (Wilcoxon rank-sum test ***  =  P<0.001).

For ON-S A-type RGCs, the median distance between PSD95CFP puncta that are nearest neighbors decreased from P12 to P21 (Figure 7A). We found a similar decrease in the median distance between YFPγ2 puncta. However, the median distance between each PSD95CFP and YFPγ2 puncta was already established at P12. In order to determine if these local relationships could be due to a random arrangement of synaptic sites onto the RGC dendrites, we calculated the nearest neighbor distributions of simulated ON-S A-type RGCs by randomly placing puncta (same densities as measured for each cell) across the dendritic arbor (Figure 7B, D, F, see Materials and Methods). The simulated distributions also showed a decrease in the nearest neighbor distances for PSD95CFP and for YFPγ2 across ages. However, the median distance between neighboring PSD95CFP and YFPγ2 puncta in the simulated distribution was greater than the imaged populations (Figure 7B, D, F). For OFF-S A-type and bistratified DS RGCs, the relative spatial distribution of excitatory and inhibitory postsynaptic puncta was also already present at P12. However, the median distances between nearest neighbor PSD95CFP and nearest neighbor YFPγ2 puncta were unchanged between P12 to P21 for both these cell types (Figure 7C, E).

### YFPγ2 and PSD95CFP puncta correspond respectively to amacrine and bipolar cell synapses on an individual RGC

**Figure 8:**
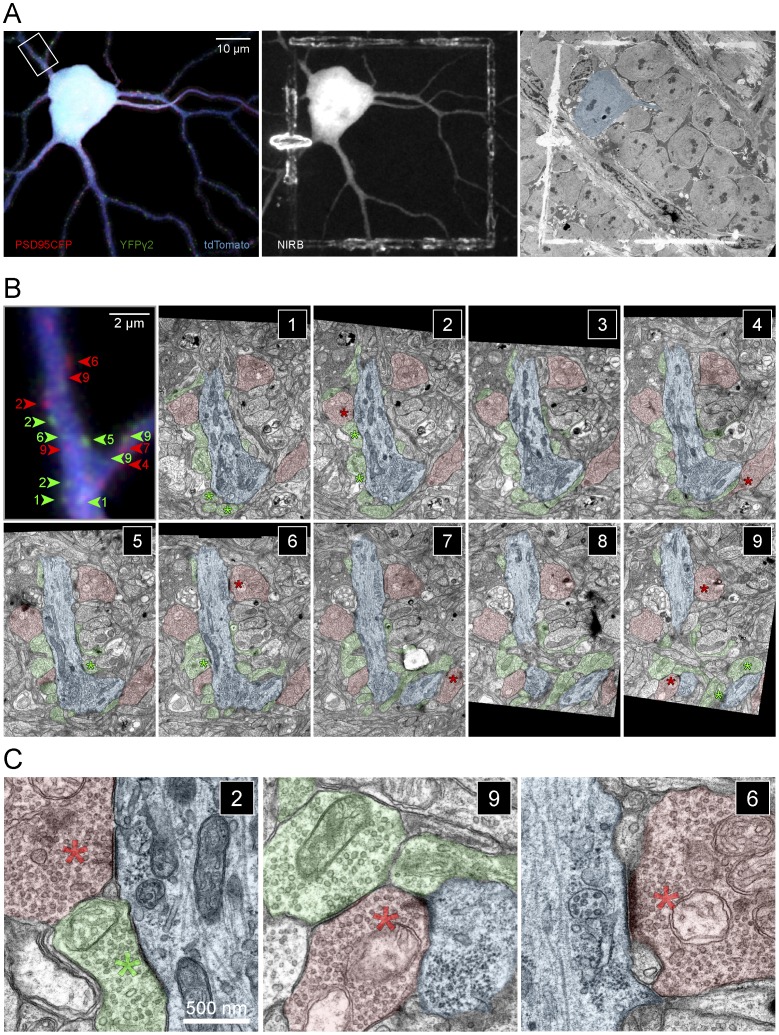
Correlation of fluorescent labeled GABAergic and glutamatergic postsynaptic sites with ultrastructure. (**A**) (Left) MIP of an ON-S A-type RGC labeled by biolistic transfection of tdTomato and PSD95CFP in a *Thy1-YFPγ2* retina. Shown here are PSD95CFP and YFPγ2 signal within the dendrites. (Middle) Image showing fiduciary marks burned at the level of the axon and cell body of the labeled RGC using the NIRBing technique [32]. (Right) EM micrograph showing the NIRBed window, allowing identification of the cell (colored blue). (**B**) MIP of a small section of a primary dendrite from the box in (A, left) showing YFPγ2 (green arrowheads) and PSD95CFP (red arrowheads) signal, and serial EM sections 1–9 of this region. Colored numbers in MIP indicate the serial section in which the corresponding synapse was identified (see green and red asterisks). EM sections are 80 nm thick. (**C**) Magnified views of bipolar (red) and amacrine (green) cell synapses on the RGC dendrite (blue) in sections (2,9,6). (Left) A cone bipolar cell terminal (red) makes a non-dyad ribbon synapse with the RGC dendrite, where two ribbons appear at the same synaptic site (red asterisk). An inhibitory amacrine cell synaptic profile can also be observed in close proximity (green asterisk). (Middle) A characteristic dyad ribbon synapse (red asterisk) between a cone bipolar cell and the RGC dendrite and an amacrine cell process (green). (Right) A contact between the ganglion cell and a bipolar cell at a plane where the postsynaptic density is evident but the ribbon is not orthogonally aligned. The synapse can be identified by examining the serial profiles in which a partial ribbon is visible (sections 4–7 in B; Movie S1).

We wished to gain a better understanding of the local synaptic connectivity associated with GABAergic and glutamatergic postsynaptic sites identified using fluorescent markers, and to determine how well these sites correlated with amacrine and bipolar cell input onto the RGC. To this end, we performed serial electron microscopy (EM) on the dendritic arbors of an ON-S A-type RGC labeled with tdTomato and PSD95CFP in a *Thy1-YFPγ2* transgenic retina (Figure 8).

To identify the labeled RGC (Figure 8A, left) under EM, we used the near infrared branding (NIRB) method [32], whereby fiduciary marks are burned into the fixed tissue using a multiphoton pulsed infrared laser (Figure 8A, middle, NIRB). These burn marks could then be identified in semi-thin resin sections of the tissue prepared for EM (Figure 8A, right). After identification of the labeled RGC, we followed the dendritic arbors through serial sections, and identified bipolar and amacrine cell synaptic profiles making contact with the RGC dendrite (Figure 8B). Synapses from bipolar cells were characterized by the presence of a presynaptic ribbon apposed to a postsynaptic density (PSD) (Figure 8C; see Movie S1). Amacrine cell synapses were identified by the presence of PSDs with no ribbons [34]. Often amacrine cell presynaptic terminals also contained dense core vesicles (data not shown). An example of the correspondence between bipolar cell synapses and PSD95CFP puncta, and between amacrine cell synapses and YFPγ2 puncta obtained by comparing the fluorescence images with the serial EM sections is provided in Figure 8B.

**Figure 9:**
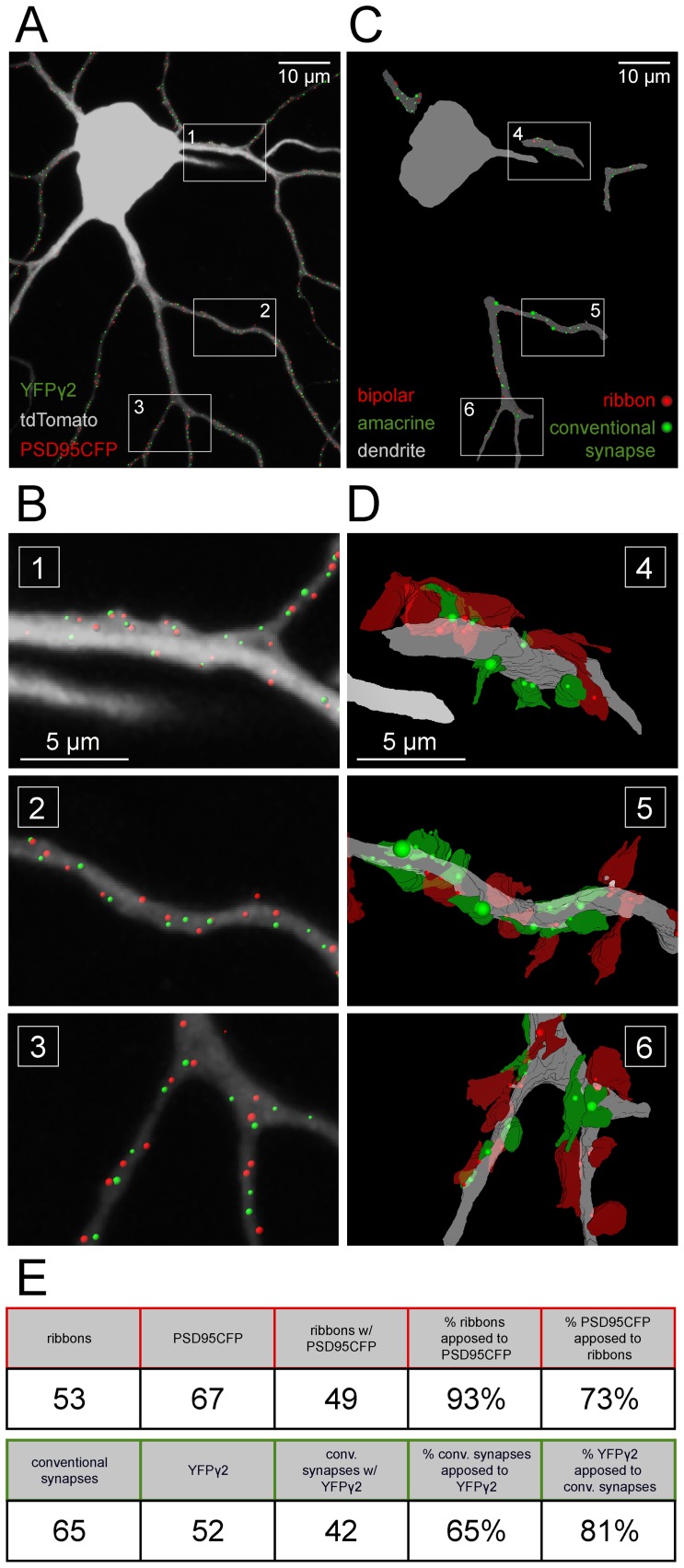
PSD95CFP and YFPγ2 puncta on RGC dendrites correspond to sites of bipolar cell and amacrine cell synapses. (**A**) ON A-type RGC (gray) with identified PSD95 (red dots) and YFPγ2 (green dots). (**B**) Higher magnification of the boxed regions 1–3 in (A). (**C**) Three dimensional reconstructions of serial EM sections showing the locations of amacrine (conventional) and bipolar cell (ribbon) synapses on the reconstructed dendrites (gray). (**D**) Higher magnification of the boxed regions in **(C)** showing amacrine (red) and bipolar (green) cell processes apposed to the dendrite. Locations of synapses are marked by the colored dots (red, ribbon; green, conventional). The size of the dot is proportional to the length of the postsynaptic density at the synapse. (**E**) Table representing the number and correlation of fluorescence and anatomical profiles from the reconstructions.

To determine how reliable is our identification of GABAergic and glutamatergic postsynaptic sites on the RGC dendritic arbors by the fluorescence labeling, we quantified the proportion of synapses on primary, secondary, and tertiary dendrites of the RGC that was identified in both confocal and serial EM reconstructions (Figure 9A–C). The high correspondence of bipolar cell ribbon synapses and amacrine cell synapses found in EM serial sections that were apposed to sites of PSD95CFP (>70% apposed to ribbons) or YFPγ2 puncta (>80% apposed to amacrine contact), respectively, strongly suggests that the fluorescently tagged postsynaptic proteins reliably mark their respective postsynaptic sites on the RGCs (Figure 9C). We believe that these numbers represent lower limits because we counted all of the fluorescent puncta within the total length of dendrite visualized from our serial reconstructions, but in the EM reconstructions we were not always able to capture the full volume of the dendrite of interest and its presynaptic contacts. Furthermore, we often found ribbon synapses only visualized in single serial sections or with irregular profiles, which could contribute to an under estimate of actual numbers [36]. The high correspondence between the position of ribbon synapses observed under EM and sites of PSD95CFP (93%) suggests that of the ribbon synapses we were able to detect, most were also labeled by a PSD95CFP puncta in confocal reconstructions. The number of conventional synapses we detected in EM that corresponded to YFPγ2 puncta was lower (65%). This may not be surprising because amacrine profiles under EM can represent GABAergic or glycinergic synapses, both of which are present on ON-S A-type RGCs [50].

Our partial EM reconstruction also allowed us to obtain a small sample of the local connectivity of excitatory and inhibitory synapses on the RGC. We found examples where single bipolar cell varicosities made multiple ribbon contacts onto the dendrite (Figure 8B), characteristic of the predominant type of bipolar cell (Type 6) that can form more than one ribbon release site onto the ON-S A-type RGC [51], [52]. We were also able to observe characteristic bipolar cell ribbon synapses in the retina, which often occur at a dyad junction [34] (Figure 8C). Indeed, we found that 75% (40 of 53) of the ribbon synapses could accurately be identified as dyad synapses. When it was possible to determine the identity of the partners (33 of 40), we found that the composition of these dyad synapses were most often onto an amacrine cell and the RGC (79%, 26 of 33), with fewer dyads comprising two RGCs (24%, 8 of 33).

As in many CNS circuits, one form of interaction between inhibitory and excitatory synapses onto a postsynaptic target in the retina is feedforward inhibition [53]. Retinal bipolar cells synapse onto amacrine cells, which in-turn can make close connections onto ganglion cells, and provide local feedforward inhibition [35]. We were able to find instances of such inhibition in our EM reconstructions. Of the dyad synapses making connections onto an amacrine cell and a ganglion cell, we found that 42% (11 of 26) made feedforward connections within the local reconstructed regions. Of the feedforward synapses, the median distance between the bipolar cell ribbon synapse and amacrine cell synapse (∼675 nm) was similar to the median distance detected using fluorescence markers (Figure 7A). Furthermore, the median nearest neighbor distances between bipolar cell ribbon synapses (∼936 nm) and between amacrine cell synapses (∼970 nm) were also similar to those quantified from fluorescence images (Figure 7A). Together, these observations suggest that our use of fluorescence markers is predictive of the connective patterns of bipolar and amacrine cell contacts on RGCs, and furthermore that many of the excitatory puncta in close proximity to inhibitory puncta found on RGC dendrites could reflect local feedforward circuits.

## Discussion

### Development of glutamatergic and GABAergic synapses on individual RGCs

Like other CNS circuits, inhibitory synaptogenesis precedes excitatory synaptogenesis in the retina [54]. Conventional synapses from amacrine cells are present shortly after birth in mouse, and increase in number at a constant rate until P21. However, ribbon synapses, characteristic of bipolar cells, are not present until P11, after which they also increase at an equivalent rate [18]. Indeed, functional drive from amacrine cells develops prior to glutamatergic transmission from bipolar cells [55]. Our previous studies separately mapping excitatory and inhibitory postsynaptic sites on individual RGCs also suggested that inhibitory and excitatory synaptogenesis may be coordinated during development to balance inhibition and excitation after glutamatergic synaptogenesis begins, at least for one RGC type [15], [26]. Indeed, such balance occurs for cultured hippocampal neurons despite increases in both inhibitory and excitatory synapse densities over time [56], [57].

We compared I/E ratios for ON-S and OFF-S A-type RGCs [28], [50] because of their similar morphologies but opposite stratification and function, and bistratified DS RGCs because of the well-defined functional role of GABAergic inhibition in bistratified DS RGCs [58]–[61]. We found that the I/E ratio is set early in development for ON-S and OFF-S A-type RGCs, before eye-opening. This is consistent with previous results in mouse where the densities of excitatory and inhibitory synaptic sites were mapped separately onto ON monostratified RGCs [15], [26]. However, bistratified DS RGC types only attained their mature I/E ratio after eye opening. The differential maturation rates across RGC types may seem in contrast to the parallel increase in amacrine and bipolar cell synaptogenesis suggested from past EM studies. However, amacrine cells also make connections with each other and with bipolar cells [62]. Thus, differential synapse maturation rates may be obscured when pooling all amacrine synapses within the IPL.

How the mature I/E ratios contribute to the functional output of RGCs has yet to be determined. It is known that the mature frequencies of spontaneous inhibitory and excitatory postsynaptic currents in individual developing RGCs are not attained until several weeks after birth, but at each developmental age they appear balanced [63]. Furthermore for ON and OFF RGCs both their spatial and temporal responses [20] and inhibitory and excitatory postsynaptic currents [19] in response to light stimuli are not mature until the third of four postnatal week. Our results suggest that the density of inhibitory and excitatory synapses on individual RGCs is not mature until after eye opening. It is intriguing to note that for the DS RGC, the strength of inhibitory input underlying the null direction increases after eye opening as direction selectivity becomes well established [64]. We observed that the density of GABAergic synapses on both the ON and OFF arbors of the bistratified DS RGC also matures after eye opening, paralleling the time course of increasing inhibition strength [64].

### Global spatial relationships of inhibitory and excitatory synapses

Few studies have measured the distributions of inhibitory and excitatory inputs onto the same neuron, particularly in tissue. Serial EM studies previously demonstrated that: (i) both apical and basal dendrites of a CA1 pyramidal neuron exhibit a pronounced proximal to distal I/E gradient of >30 fold [5], and (ii) the I/E ratios of three distinct types of hippocampal interneurons changed several fold across their arbors [8]. In contrast, we found that the I/E ratios of three RGC types do not vary greatly across ≥80% of their dendritic field. This may be because unlike pyramidal neurons that receive distinct types of excitatory input onto their apical and basal dendrites [3], [65], inputs from a given bipolar cell type are distributed across the entire arbor of a RGC [52]. While inhibitory synapses vary greatly in density across the pyramidal neuron's arbor, reflecting contacts from diverse populations of inhibitory interneurons [65], we found, instead, that GABAergic synapse density is relatively unchanged across the RGC arbor. It remains possible that synapses of a specific amacrine cell type are differentially localized across the RGC arbor, but it is not yet known which inhibitory amacrine cell types provide GABAergic input onto all RGC types. For DS RGCs where the primary presynaptic amacrine cell types are known, there is uniform distribution of amacrine contacts across the DS dendritic arbors [66], [67]. The relatively even distributions of GABAergic and glutamatatergic synapses onto RGCs may be a common result of bipolar and amacrine cells forming mosaics across the retina [68], [69].

Although previous fluorescence imaging studies reported little difference in the distributions of excitatory postsynaptic sites across different RGC types [48], [70], this may not be true for all types [26]. Immunohistochemical markers for synaptic proteins also show that I/E ratios are more evenly matched across RGC types in marmoset [71], [72], but EM reconstructions revealed considerable variation in the I/E ratio across RGC types of multiple species [73]–[76]. We found that mature I/E ratios can vary across mouse RGC types with respect to GABAergic and glutamatergic synapses.

How does the overall distribution of synaptic inputs onto individual neurons inform us of cell function? At least for some RGCs, their functional properties can be predicted by the distribution of their inputs [26], [77], but this is not true for other RGCs. The ‘preferred direction’ of direction-selective RGCs is a consequence of the circuit properties of their presynaptic inhibitory input [58], [59], [61], [67] rather than the spatial distribution of their inhibitory inputs [66]. Together with the excitatory and inhibitory synapse maps we provide here, future identification of the presynaptic partners, their functional properties and their connectivity should lead to a more complete understanding how the output of distinct RGC types are shaped.

### Local spatial relationships between GABAergic and glutamatergic synapses onto RGC

Functional studies that directly examined the spread of integration between synaptic inputs suggest that synaptic integration may be localized to dendritic segments spanning as little as 10 µm [78]–[80], and for spiny neurons, to spines [81]. However, much of this work focused primarily on the interaction of neighboring excitatory inputs. Modeling studies suggested that inhibitory inputs further the computational abilities of individual dendrites [82], [83]. Moreover, cell culture studies demonstrated the capacity for inhibitory inputs to affect excitatory inputs with spatial constraints similar to those seen between excitatory inputs [84], perhaps representing subcircuit arrangements. The axonal territories of most bipolar cell types are relatively small (10–25 µm diameter) and tile across a RGC dendritic arbor [69] such that bipolar cell inputs likely represent repeated subcircuits. Inhibitory input onto RGCs may locally regulate excitation within these subcircuits across the RGC receptive field. Indeed, a key synaptic arrangement within the retina is the ribbon dyad synapse of bipolar cells, whereby a single bipolar cell release site is apposed to two processes [34]. Amacrine cells have been found to directly synapse onto the RGC at these dyad synapses, thus providing local feedforward inhibition. In primate, local feedforward inhibition is found at more than half of the amacrine cell contacts onto one RGC type [35] and is important for cell function [85]. But, these feedforward circuits may not dominate in all RGC types [72]. Our EM observations suggest that for the ON-S A-type RGC in the mouse retina, ∼42% of the dyad contacts sampled have local feedforward inhibition. We also found that the median distances between fluorescently labeled excitatory and inhibitory postsynaptic sites resemble the distance between the bipolar cell contact and the amacrine cell feedforward synapse onto the RGC dendrite. This was true for all RGC types examined. Although we cannot conclude that these synapse arrangements visualized under light microscopy always reflect subcircuits comprising feedforward inhibition, we showed that the local spatial arrangements cannot occur merely by random organization of synaptic sites onto the RGC. Thus, there may exist local constraints on the spatial relationship between sites of glutamatergic excitation and GABAergic inhibition onto RGC dendritic arbors that favor local feedforward circuits.

In the primate retina, dyad synapses have been suggested to form from an initial monad contact between a bipolar cell and an RGC or amacrine cell followed by addition of a contact with one of these cell types [86], [87]. Our observation that the local spatial relationship between inhibitory and excitatory postsynaptic sites are set by P12 for all three RGC types, suggest that dyads form rapidly after bipolar cells make synapses. What remains unresolved is whether the dyad arrangement sets the median distances between neighboring amacrine and bipolar inputs, or whether some cue that initially imposes a spatial constraint on this distance facilitates dyad formation. In hippocampal cell culture, disruption to inhibitory transmission results in compensatory alterations in excitatory transmission only when these inputs are present on the same dendritic branch [84]. In addition, two recent studies have shown that alterations in excitatory activity during development result in decreases in a specific subset of inhibitory synapses next to excitatory inputs on dendritic spines [6], [7]. The stereotypic synaptic dyad arrangement of inputs onto RGC dendrites clearly presents an accessible model for future work aimed at elucidating the mechanisms responsible for setting up local circuit architecture necessary for balancing inhibitory and excitatory input.

## Supporting Information

Figure S1 **Dependence of cross-correlation analysis on differences in intensity and signal to noise.** **(A)** Demonstration of how differences in intensity between two channels affects the correlation coefficient. A 10×15×10 µm (xyz) volume from P21 vertical retinal slices immunostained with gephyrin (channel 1 column), an inhibitory postsynaptic scaffolding protein [88], [89], is correlated with a duplicated volume (channel 2). In the duplicated channel, the percent intensity difference between the channels was varied from 1–99 by linearly scaling the intensity of the duplicate channel (channel 2 column). The correlation coefficient at each z-depth (µm) is relatively unchanged until the intensity difference between the channels is >90% (corr. coeff column). This persistence in the correlation coefficient can best be demonstrated by observing that much of the signal-to-noise apparently lost by differences in intensity, can be recovered by rescaling the ‘dimmer’ image to the full bit depth (2 rescaled column). Only at intensity differences of >90% does the rescaled channel begin to be degraded. However, even at these extremes, much of the spatial patterns between the two channels are preserved. (**B**) To determine how changes in signal-to-noise between two fluorescence channels affect the correlation coefficient, the same immunostained volume (channel 1 column) was correlated with its duplicate when the percentage of voxels is systematically scrambled from 1–99 (channel 2 column). The strength of the correlation coefficient at each depth follows inversely the percent of scrambled voxels (corr. coeff column). (**C**) Plot of the average correlation coefficient vs changes in intensity (red) or scrambled voxels (green) at each interval.
(TIF)

Figure S2 **Stratification of ON-S A-type, OFF-S A-type and bistratified DS RGCs can be readily identified in** ***Thy1-YFPγ2*** **retinas.** (**A, B**) Vertical sections from P12 and P21 retinas immunostained with anti-VAChT to label the presynaptic terminals of starburst amacrine cells [90], [91]. These characteristic cholinergic bands (arrow heads in A–C) can be utilized to identify stratification in the retina. Upper panels are maximum intensity projections (MIPs) of image stacks of 15 µm total thickness where two bands of relatively dense YFPγ2 fluorescence correspond to the VAChT positive sublaminae. The high degree of spatial apposition between VAChT staining and YFPγ2 fluorescence is apparent in single optical sections (0.3 µm) within the stack (lower panels). (**C, D**) Examples of vertical MIPs of ON-S, OFF-S A-type and bistratified DS RGCs labeled with tdTomato at P12 and P21. At both ages, the dendritic arbors of ON-S A-type RGCs consistently stratified below the lower VAChT band, whereas the arbors of OFF-S A-type RGCs stratified above the upper VAChT band. The arbors of bistratified DS RGCs costratify with the VAChT bands. Scale bars are 5 µm.
(TIF)

Figure S3 **Only a fraction of the cells in the ganglion cell layer express YFPγ2.** (**A**) MIP of an image stack encompassing the ganglion cell layer from a P21 mouse. TO-PRO-3 staining reveals the nuclei of all cells in the ganglion cell layer (red), and cell bodies expressing YFPγ2 can be easily identified (gray). (**B**) Quantification of the density of TO-PRO-3 labeled cells, and YFPγ2 expressing cells within a 235 µm^2^ sampled region (n = 4 retinas, 7 regions). (**C**) Cells expressing YFPγ2 represent 21.8% of the cells in the ganglion cell layer. (**D**) Vertical sections from a P21 retina immunostained with anti-GABA_A_R γ2. Upper panels are MIPs of image stacks of 12 µm total thickness. Lower panels show single optical sections (0.3 µm) within the stack at higher magnification. Note that whereas all YFPγ2 puncta are also labeled by anti-GABA_A_R γ2, not all anti-GABA_A_R γ2 have YFPγ2.
(TIF)

Movie S1 **Serial walk-through of bipolar cell ribbon contacts with a RGC dendrite.** Serial sections through the synaptic contact between the bipolar cell axon terminal (red) and the RGC dendrite (blue) highlighted in Figure 8B, C panel 6. Two ribbon synapses can be seen (arrowheads): 1) A ribbon synapse parallel to the plane of sectioning and 2) a ribbon synapse orthogonal to the plane of sectioning. Both forming dyad synapses with the RGC and a dendritic process from a different cell.
(MOV)
