# Supplementary figures and images for: Spatial Relationships between GABAergic and Glutamatergic Synapses on the Dendrites of Distinct Types of Mouse Retinal Ganglion Cells across Development
Source: PLoS One. 2013 Jul 26;8(7):e69612. doi: 10.1371/journal.pone.0069612 (PMC3724919; doi:10.1371/journal.pone.0069612)

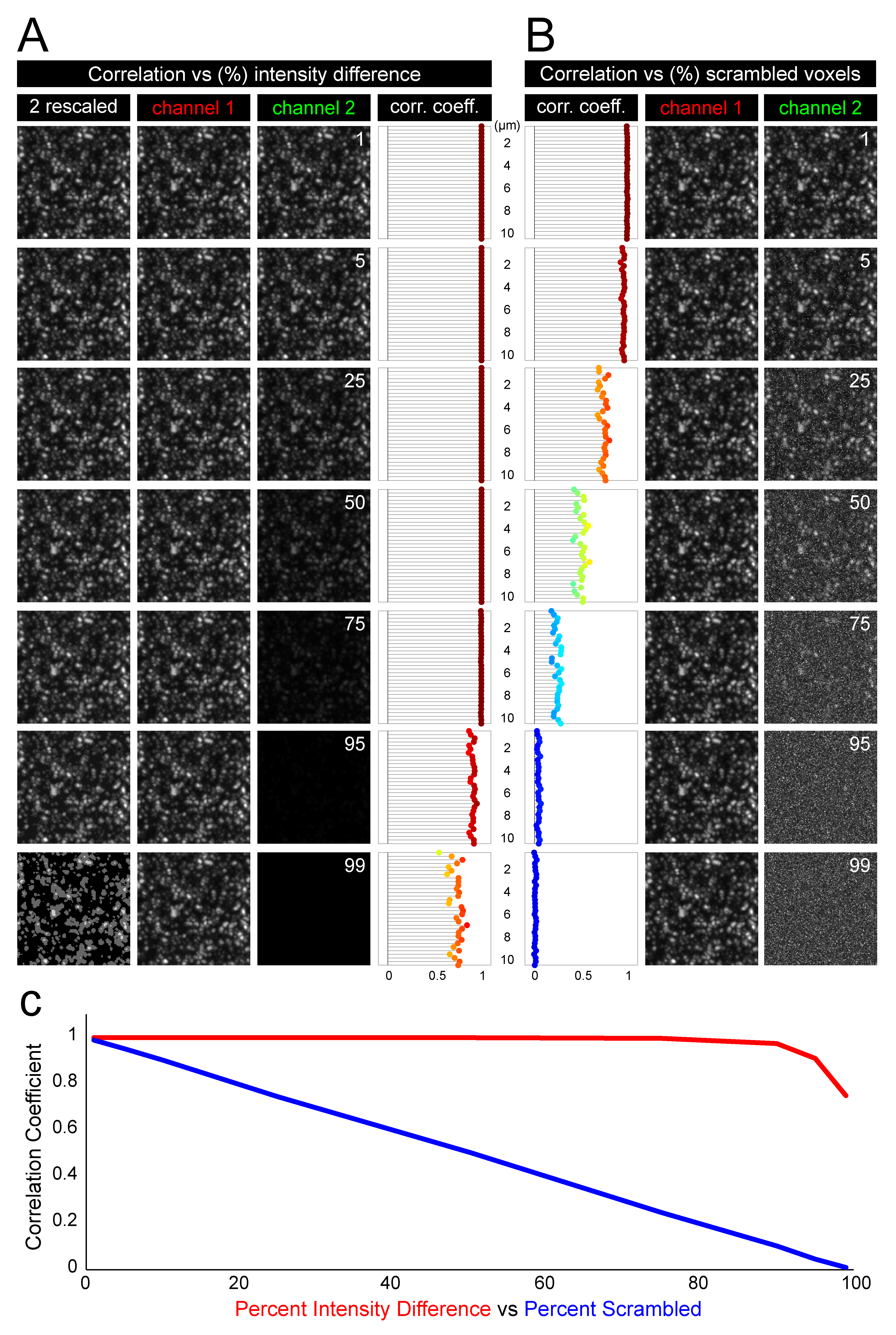

Supplement: Figure S1 — Dependence of cross-correlation analysis on differences in intensity and signal to noise. (A) Demonstration of how differences in intensity between two channels affects the correlation coefficient. A 10×15×10 µm (xyz) volume from P21 vertical retinal slices immunostained with gephyrin (channel 1 column), an inhibitory postsynaptic scaffolding protein [88], [89], is correlated with a duplicated volume (channel 2). In the duplicated channel, the percent intensity difference between the channels was varied from 1–99 by linearly scaling the intensity of the duplicate channel (channel 2 column). The correlation coefficient at each z-depth (µm) is relatively unchanged until the intensity difference between the channels is >90% (corr. coeff column). This persistence in the correlation coefficient can best be demonstrated by observing that much of the signal-to-noise apparently lost by differences in intensity, can be recovered by rescaling the ‘dimmer’ image to the full bit depth (2 rescaled column). Only at intensity differences of >90% does the rescaled channel begin to be degraded. However, even at these extremes, much of the spatial patterns between the two channels are preserved. (B) To determine how changes in signal-to-noise between two fluorescence channels affect the correlation coefficient, the same immunostained volume (channel 1 column) was correlated with its duplicate when the percentage of voxels is systematically scrambled from 1–99 (channel 2 column). The strength of the correlation coefficient at each depth follows inversely the percent of scrambled voxels (corr. coeff column). (C) Plot of the average correlation coefficient vs changes in intensity (red) or scrambled voxels (green) at each interval. (TIF) [file pone.0069612.s001.tif]

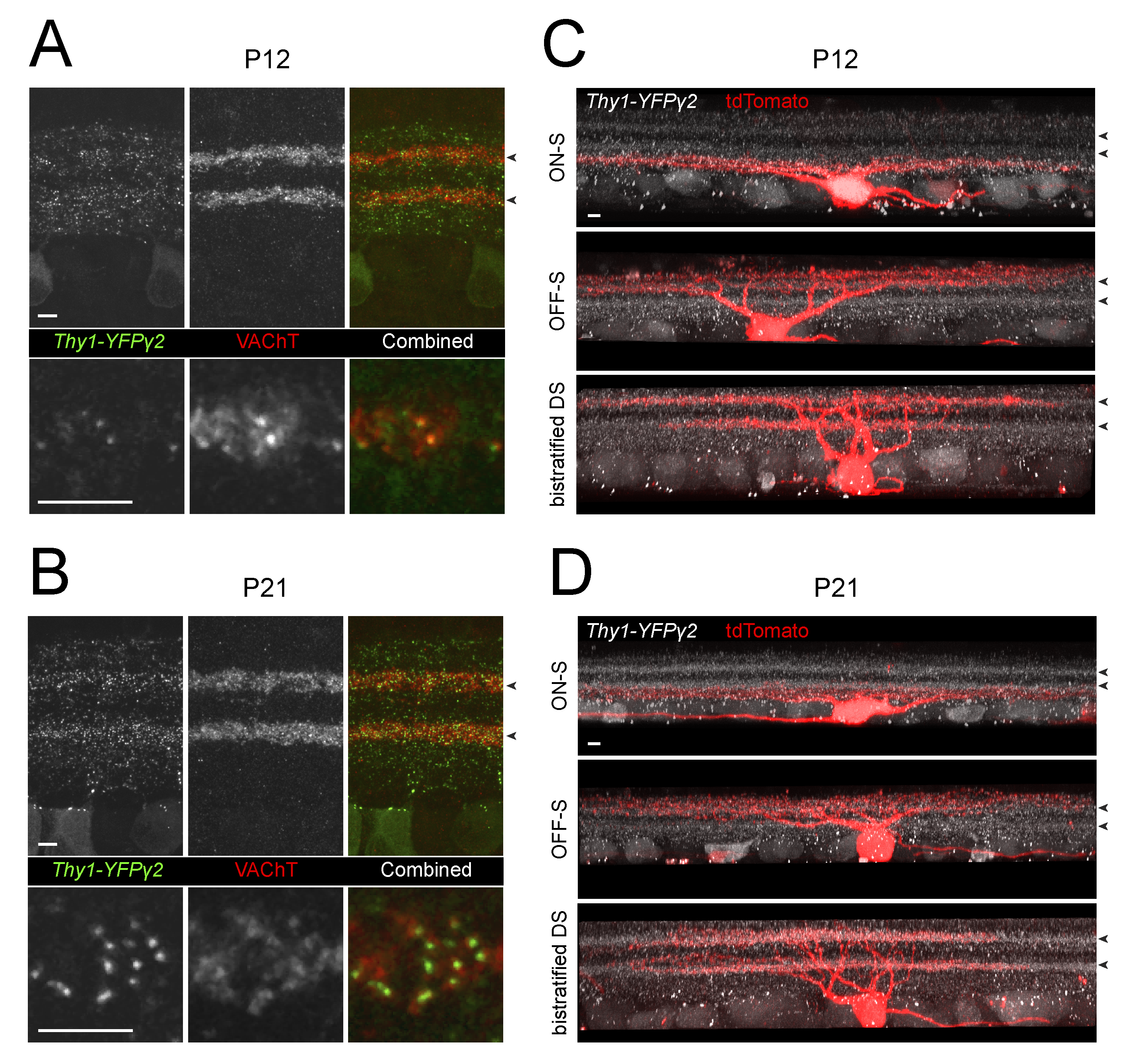

Supplement: Figure S2 — Stratification of ON-S A-type, OFF-S A-type and bistratified DS RGCs can be readily identified in Thy1-YFPγ2 retinas. (A, B) Vertical sections from P12 and P21 retinas immunostained with anti-VAChT to label the presynaptic terminals of starburst amacrine cells [90], [91]. These characteristic cholinergic bands (arrow heads in A–C) can be utilized to identify stratification in the retina. Upper panels are maximum intensity projections (MIPs) of image stacks of 15 µm total thickness where two bands of relatively dense YFPγ2 fluorescence correspond to the VAChT positive sublaminae. The high degree of spatial apposition between VAChT staining and YFPγ2 fluorescence is apparent in single optical sections (0.3 µm) within the stack (lower panels). (C, D) Examples of vertical MIPs of ON-S, OFF-S A-type and bistratified DS RGCs labeled with tdTomato at P12 and P21. At both ages, the dendritic arbors of ON-S A-type RGCs consistently stratified below the lower VAChT band, whereas the arbors of OFF-S A-type RGCs stratified above the upper VAChT band. The arbors of bistratified DS RGCs costratify with the VAChT bands. Scale bars are 5 µm. (TIF) [file pone.0069612.s002.tif]

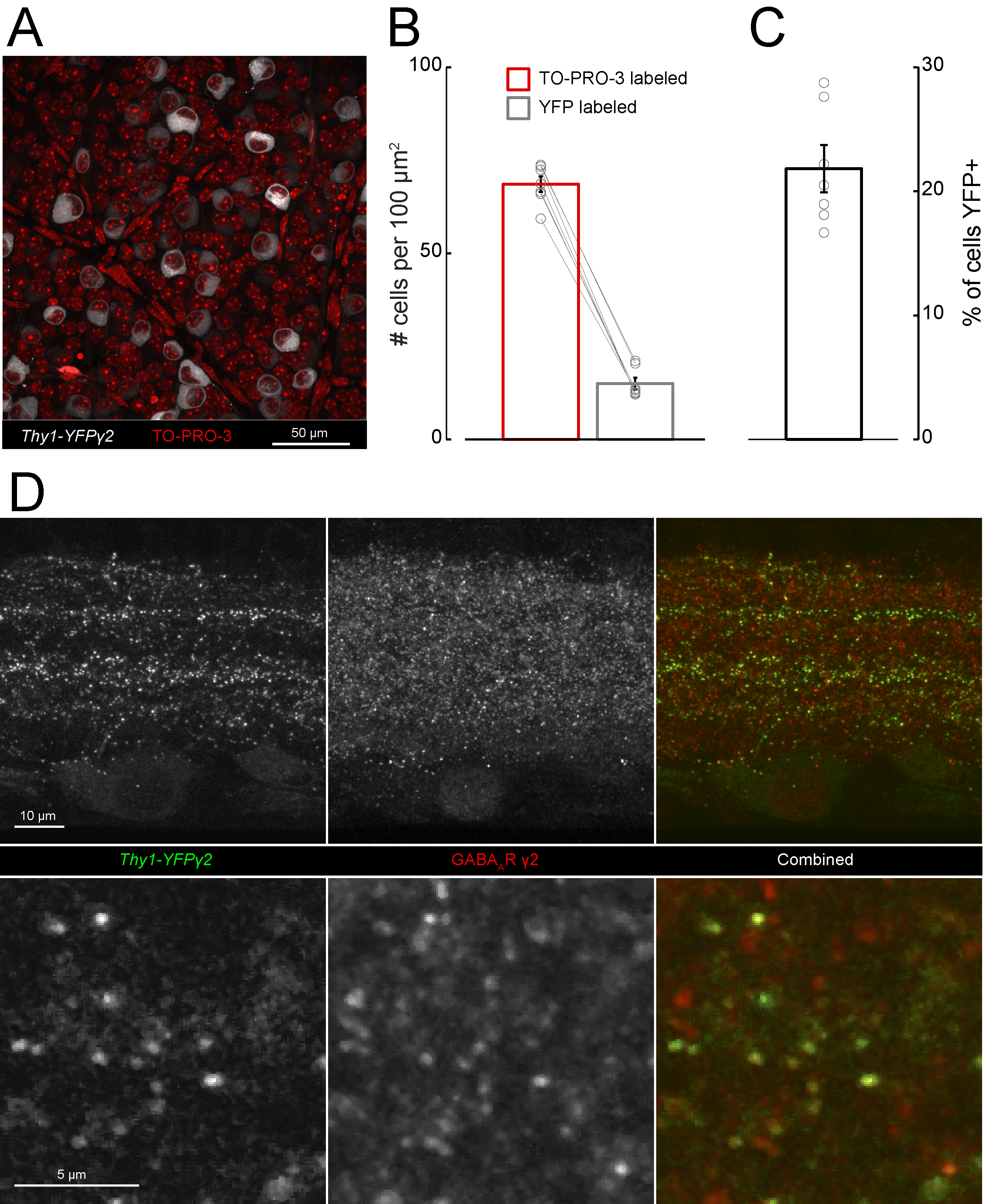

Supplement: Figure S3 — Only a fraction of the cells in the ganglion cell layer express YFPγ2. (A) MIP of an image stack encompassing the ganglion cell layer from a P21 mouse. TO-PRO-3 staining reveals the nuclei of all cells in the ganglion cell layer (red), and cell bodies expressing YFPγ2 can be easily identified (gray). (B) Quantification of the density of TO-PRO-3 labeled cells, and YFPγ2 expressing cells within a 235 µm2 sampled region (n = 4 retinas, 7 regions). (C) Cells expressing YFPγ2 represent 21.8% of the cells in the ganglion cell layer. (D) Vertical sections from a P21 retina immunostained with anti-GABAAR γ2. Upper panels are MIPs of image stacks of 12 µm total thickness. Lower panels show single optical sections (0.3 µm) within the stack at higher magnification. Note that whereas all YFPγ2 puncta are also labeled by anti-GABAAR γ2, not all anti-GABAAR γ2 have YFPγ2. (TIF) [file pone.0069612.s003.tif]
